# Characterizing Factors Associated with Excess Body Weight: A Descriptive Study Using Principal Component Analysis in a Population with Overweight and Obesity

**DOI:** 10.3390/nu16081143

**Published:** 2024-04-12

**Authors:** Álvaro Fernández-Cardero, José Luis Sierra-Cinos, Adrián López-Jiménez, Beatriz Beltrán, Carmen Cuadrado, María Teresa García-Conesa, Laura Bravo, Beatriz Sarriá

**Affiliations:** 1 Department of Metabolism and Nutrition, Institute of Food Science, Technology and Nutrition (ICTAN), Spanish National Research Council (CSIC), C/Jose Antonio Novais 6, 28040 Madrid, Spain; alvafe22@ucm.es (Á.F.-C.); adrianlopezjimenezz@gmail.com (A.L.-J.); lbravo@ictan.csic.es (L.B.); 2 Department of Nutrition and Food Science I, School of Pharmacy, Universidad Complutense de Madrid, Ciudad Universitaria s/n, 28040 Madrid, Spain; joselsie@ucm.es (J.L.S.-C.); beabel@ucm.es (B.B.); ccuadrad@ucm.es (C.C.); 3 Department of Health Science, School of Health Science, Universidad International Isabel I de Burgos (Ui1), C. de Fernán González, 76, 09003 Burgos, Spain; 4 Department of Food Science and Technology, Centro de Edafología y Biología Aplicada del Segura (CEBAS), Spanish National Research Council (CSIC), Campus de Espinardo, 30100 Murcia, Spain; mtconesa@cebas.csic.es

**Keywords:** overweight, obesity, inter-individual variability, cardiometabolic risk, factor analysis, dietary intake, polyphenols, body composition, physical activity, energy expenditure

## Abstract

Obesity is a worldwide epidemic, making it crucial to understand how it can be effectively prevented/treated. Considering that obesity is a multifactorial condition, this article carried out a baseline cross-sectional study of the variables involved in the disorder. Eighty-four subjects with overweight/obesity were recruited. Dietary baseline information was obtained by analysing three 24 h recalls. Resting metabolic rate was measured using indirect calorimetry, physical activity was measured through accelerometry, cardiometabolic parameters were determined in blood samples and body composition via anthropometry and bioimpedance. A univariant and multivariate exploratory approach was carried out using principal component analysis (PCA). Large inter-individual variability was observed in dietetic, biochemical, and physical activity measurements (coefficient of variation ≥ 30%), but body composition was more uniform. Volunteers had an unbalanced diet and low levels of physical activity. PCA reduced the 26 analysed variables to 4 factors, accounting for 65.4% of the total data variance. The main factor was the “dietetic factor”, responsible for 24.0% of the total variance and mainly related to energy intake, lipids, and saturated fatty acids. The second was the “cardiometabolic factor” (explaining 16.8% of the variability), the third was the “adiposity factor” (15.2%), and the last was the “serum cholesterol factor” (9.4%).

## 1. Introduction

Although there is an open debate as to whether obesity should be considered a disease [1], it is undeniable that excessive body fat accumulation, including both overweight and obesity, have reached worldwide epidemic proportions in recent decades, as highlighted by the World Health Organization (WHO, fact sheet 311) [2,3]. Worldwide, obesity has nearly tripled since 1975, despite public health policies and treatment efforts to combat the obesity epidemic [4]. Estimations from 2016–2017 projected that if the increasing trend in obesity continued, by 2025, the global prevalence of obesity will reach 20% of the population [5]. However, these figures were surpassed in Europe in 2022 [3].

Far more than an imbalance between food intake and energy expenditure, obesity is a complex multifactorial disorder resulting from the interaction of a range of conditions, including lifestyle (mostly diet and physical activity), social, and environmental aspects (obesogenic environment, country, religion, socioeconomic status, and education), as well as biological factors (age, sex, genotype, epigenetic modifications, and microbiota composition), among others [6].

For many years, dietary strategies to lose weight have consisted of varying macronutrient composition (e.g., low-carbohydrate or low-fat diets) as well as restricting total energy (e.g., low-calorie, intermittent fasting) intake, while a reduction in body weight and body mass index (BMI) have been the main targets used to determine the effectiveness of the weight reduction treatment. However, there has been a progressive shift in the approach to weight loss strategies and the focus on relevant endpoints [7]. For instance, BMI has been a population-level measure of overweight and obesity that was commonly used for both sexes and adults of all ages; however, this index has also been widely criticised, as BMI varies greatly among individuals based on age, sex, and ethnicity. Furthermore, BMI is not sensitive enough to differentiate the level or distribution of adipose tissue mass and the different health-related implications. Indeed, for any given amount of body fat, greater cardio-metabolic risk has been associated with the localization of excess fat in the visceral adipose tissue and ectopic depots (such as the muscle, liver, and pancreas) [8,9].

Additionally, it is important to consider improvements in health as a relevant endpoint in weight loss treatments. While many types of dietary interventions can achieve a negative energy balance, a successful dietary strategy permits a loss of at least 5% of the baseline body weight along with improvement in cardiometabolic health outcomes [7]. Given the large variability between individuals in their responses to most weight loss treatments, it is essential that future research addresses the causes of this variability. Thus, it is important to incorporate more rigorous analyses of the key factors involved in this variability, such as dietary composition and dietary patterns and energy requirements and energy expenditure (e.g., resting metabolic rate and energy expenditure from physical activity) [10].

To further increase the success of weight-loss treatments, excess body weight must be addressed with a multidisciplinary approach. Sex and age are important factors in the individualization process due to critical differences in hormone concentration, adiposity, fat distribution [11,12], and microbiota composition [13], as well as different eating behaviours and dietary habits [14]. Demographic factors (educational level, ethnicity, nationality, income level, occupation, etc.) are also associated with obesity at individual and community levels; thus, understanding the relationship between these factors is essential to implement changes/recommendations that may help to fight the obesity epidemic [15]. In this context, nutritional advice based on the unique characteristics of an individual has emerged as a new strategy to improve the way obesity is tackled. Personalized nutrition tries to take many of these multiple factors into account based on baseline information to then make decisions and choose the most appropriate weight loss treatment [16,17]. More research must be conducted to determine distinguishable features for subsequent risk and response stratification [1].

The purpose of the current article is to thoroughly understand the baseline characteristics related to overweight/obesity in the study population enrolled in the GREENCOF project by analysing the common variation between the original variables involved in the disorder, and then condensing the large dataset into a few derived variables. To achieve this main purpose, a univariant and multivariate approach was carried out using principal component analysis (PCA). In order to support the variables included in the PCA, we attempted to (1) understand if there are sex-related differences in the main variables that were studied, (2) examine volunteers’ diet in order to distinguish possible dietary patterns and identify participants who misreported their energy intake, and (3) look into the inter-individual variability in the studied parameters. These points were tackled before the PCA was carried out.

## 2. Materials and Methods

### 2.1. Study Design

A cross-sectional study was carried out to obtain an in-depth description and multifaceted characterization of a cohort enrolled in a dietetic intervention under baseline conditions. Therefore, data on sex, age, ethnicity, education, socioeconomic level, anthropometric and body composition, diet, and energy expenditure will be addressed in the present article. This study corresponds to the baseline characteristics of participants in the GREENCOF nutritional intervention, which aimed to understand the effects of a coffee rich in polyphenols on overweight/obesity and its associated cardiometabolic risk, with a focus on the inter-individual variability in the responses.

For this study, men and women with overweight and grade I and II obesity (BMI between 25 and 36 kg/m^2^), between 20 and 65 years old, were recruited (inclusion criteria). Exclusion criteria were as follows: people following a vegetarian/vegan dietary pattern, smokers, pregnant or breastfeeding women, people taking dietary supplements (vitamins, antioxidants, etc.), people having taken antibiotics in the two months before the start of the study, and people having changed their lifestyle habits (diet, physical activity) in the last 6 months. People who were on a treatment with prescription drugs that did not interfere with the outcomes of the study, or that were on a long-term therapy and were well-controlled/stable, were accepted into the study.

The GREENCOF trial was registered in ClinicalTrials (NCT06204445). The protocol of the study was approved by the Clinical Research Ethics Committee of Hospital Universitario Puerta de Hierro (Majadahonda, Spain) and the Bioethics Committee of Spanish National Research Council (CSIC). Before the beginning of the study, the participants had to sign an informed consent. The study was carried out at the Human Nutrition Unit (HNU) of the Institute of Food Science, Technology and Nutrition (ICTAN), where all tests were performed.

### 2.2. Sample Size Calculation

For the GREENCOF intervention, aimed at studying the potential slimming effects of coffee on people with overweight/obesity, among other health effects. Sample size was calculated using the G Power 3.1.9.7 software taking body weight as the main variable, assuming a statistical power of 80%, a level of statistical significance of 5%, a standard deviation of 6.5, and aiming to detect a pre–post difference of 2.5 kg in a randomized, blind, cross-over intervention. Considering these premises, the sample size was estimated to be 38 subjects. However, in the GREENCOF study, inter-individual variability was addressed by considering many other overweight/obesity-related factors. Therefore, we enlarged the number of recruited volunteers and anticipated that we would have enough statistical power to detect changes in the mentioned primary outcome. In addition, the effects on other obesity-related factors could also be investigated, and subgroups of responders and non-responders to the intervention could be established.

However, the main goal of the present work is to thoroughly describe the study population for the ongoing intervention trial. Therefore, participants were recruited based on this premise, and we recruited more to analyse interindividual variability. The PCA approach was used to reduce the large number of variables into a small number of components that could explain better the correlations between data. We assessed the adequacy of data using the Kaiser–Mayer–Olkin (KMO) test and the Barlett test of sphericity (*p* < 0.001). If the KMO values were under 0.6, we considered the data to be inadequate to carry out the PCA approach due to the low sample size (https://www.ibm.com/docs/en/spss-statistics/29.0.0?topic=detection-kmo-bartletts-test, accessed on 3 March 2024). In our case, the KMO values were above 0.6 and close to 0.7 or more (0.787 in the first PCA, 0.670 in the second, and 0.677 in the third) and the *p* values of the Barlett test were below 0.001.

### 2.3. Recruitment

Participants were recruited by placing advertisements on the website of the GREENCOF study (www.proyectogreencof.es), sending many e-mails to addresses found in CSIC and Complutense University of Madrid (UCM) mail lists, and placing informative posters around the UCM campus, CSIC centres located in Madrid, cultural centres, libraries, or billboards in the area near ICTAN. Other forms of recruiting people consisted of giving short talks in classes held at the Complutense University, distributing flyers in pharmacies, organizing science dissemination activities, or using social networks to improve the distribution and the variety of people enrolled in the study. In addition, people who participated in previous human studies at ICTAN were contacted using the HNU database in ICTAN. The recruitment was also supported by the Spanish Society of Family and Community Pharmacy (SEFAC). The objective was to recruit students, employees, and a wide range of people with different backgrounds and educational levels to avoid biasing the sample of study participants. A self-reported questionnaire was distributed to the volunteers to attain information on the following sociodemographic characteristics: sex, age, monthly income, education level, and ethnicity.

**Figure 1:**
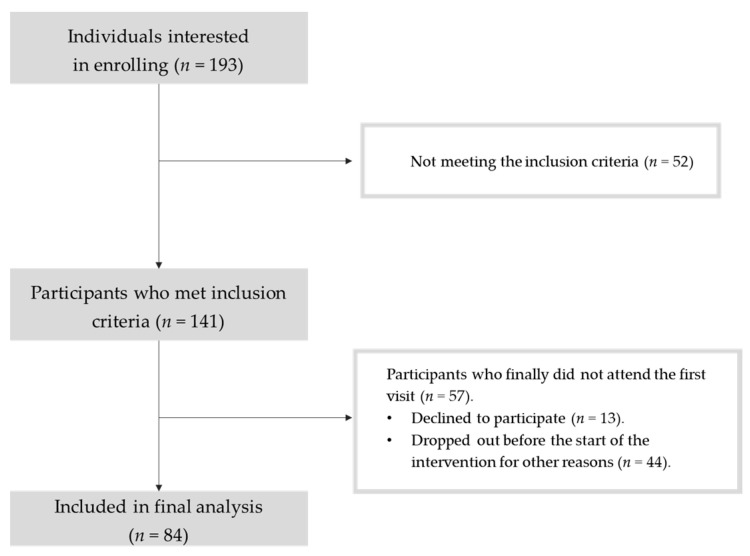
Flow chart of the recruitment process.

A flow chart of the recruitment process is shown in Figure 1.

### 2.4. Dietary Intake Measurements

Before the start of the intervention, trained scientists interviewed the participants, who filled out 24 h dietary recalls that collected all the food and beverages consumed within 24 h on three different days, including two working days and one weekend day or holiday. Energy intake, macronutrients, simple sugars (both intrinsic and added), alcohol, dietary fibre, cholesterol intake and the lipid profile of the diet (saturated fatty acids [SFA], monounsaturated fatty acids [MUFA], and polyunsaturated fatty acids [PUFA]) were calculated using the DIAL software [for Windows version 3.15.3; Department of Nutrition and Food Science (UCM) and Alce Ingeniería, S.A. Madrid, Spain]. The energy density of the diet was calculated as the ratio between energy intake (in kcal) and total edible food intake in grams.

(Poly)phenol intake was calculated using: (1) the average quantity of each food item intake (g/day) obtained from the 24 h dietary recalls analysed by the DIAL software, and (2) the corresponding content of (poly)phenols (mg/100 g), assessed with a simple tool created in a Microsoft Excel spreadsheet and data extracted from the Phenol-Explorer database (www.phenol-explorer.eu, accessed on 20 December 2023) [18,19]. Total daily (poly)phenol intake was estimated as the sum of the (poly)phenol intake from each food group, then corrected by individual energy intake and expressed as total (poly)phenol intake per 1000 kcal. More detailed information on the food groups that were included or not considered in the calculation is provided in the Supplementary Information (Table S1). It is worth noting that the subjects taking part in this study were going through a two-week coffee-washing period before starting the coffee intervention, and thus this drink was excluded from the estimation of the (poly)phenols intake.

### 2.5. Anthropometric, Body Composition, Physical Activity, and Resting Metabolic Rate Analysis

A trained anthropometrist, certificated by the International Society for the Advancement of Kinanthropometry (ISAK), took all the anthropometric measurements following ISAK recommendations and protocols to reduce interindividual errors. Participants’ heights were measured with a Holtain Harpenden precision stadiometer (Holtain Ltd., Crymych, Pebs, Wales, UK) and body weight was measured with an Omron scale (Body Composition Monitor BF511, OMRON, Hoodfddop, The Netherlands). BMI was calculated as the quotient between body weight in kilograms and squared height in metres (kg/m^2^). Waist, hip, brachial, thigh, and calf circumferences were taken with a Cescorf anthropometric tape (Porto Alegre, Brazil). Six anthropometric skinfolds (tricipital, bicipital, subscapular, suprailiac, mid-thigh, and calf) were measured using a Holtain skinfold calliper (Holtain Ltd., Crymych, Pembs, Wales, UK). Then, all six skinfolds were added up to create the variable “SUMM of 6 skinfolds” representing the subcutaneous adipose tissue.

A multi-frequency bioelectrical impedance analysis (BIA) with the INBODY S10 Body Composition Analyser (InBody Co., Ltd., Chungcheongnam-do, Korea) was used to estimate total body fat, skeletal muscle mass (SMM, both in kilograms), and visceral fat area (VFA, in squared centimetres). Skeletal muscle index (SMI) was calculated as the quotient between skeletal muscle mass and squared height in metres (kg/m^2^).

Accelerometers were used to monitor the physical activity of the participants, who wore them continuously for one week, only removing them when in contact with water (i.e., showering/bathing or at the swimming pool, in which case they noted the time spent swimming). The accelerometers (ActiGraph wGT3X-BT; CamNtech Ltd., Pensacola, FL, USA) registered high-resolution raw acceleration data in three axes, which could be transformed into other variables, such as the average physical activity expenditure per day (kcal/day), the average step count per day, or the Metabolic Equivalent of a Task (MET), where one MET was equal to 3.5 mL of oxygen per kilogram of body weight per minute (mL/kg/min). An excel sheet was generated with all these data using the ActiLife 6 software (ActiGraph version 6.13.5.). The software extracted the raw acceleration values, as well as the transformed variables (physical activity expenditure per day, average step count per day, and MET) for each participant.

To assess the Resting Metabolic Rate (RMR), indirect calorimetry was performed using the FITMATE Pro portable calorimeter (Version 4.10, Cosmed, Bicester, UK). Total energy expenditure (TEE) was calculated as RMR plus average physical activity expenditure, plus the thermogenic effect of the food (estimated as 10% of RMR). Finally, Physical Activity Level (PAL) was calculated using the rTEE:RMR ratio, using the European Food Safety Authority (EFSA) criteria. Age-specific (18–69 years) cut-off values, reported by EFSA’s scientific opinion on dietary reference values for energy (2013), were used to classify subjects according to their PAL, as follows: 1.4–1.6 was classified as low physical activity, 1.6–1.8 as moderate, and >1.8 as high physical activity [20].

### 2.6. Biochemical Analysis and Blood Pressure Measurement

Volunteers came to the HNU at ICTAN after an overnight fast. A nurse extracted fasting blood samples into tubes without an anticoagulant or EDTA-coated tubes that were centrifuged to obtain serum and plasma samples. Aliquots were kept frozen at −80 °C until analysis.

Total cholesterol (TC), high-density lipoprotein cholesterol (HDL), low-density lipoprotein cholesterol (LDL), very low-density lipoprotein cholesterol (VLDL), triglycerides (TG), fasting blood glucose (FBG), insulin, glycated haemoglobin (HbA1c), aspartate aminotransferase (AST), and alanine aminotransferase (ALAT) were measured using serum samples according to the reference methods recommended by the Spanish Society of Clinical Biochemistry and Molecular Pathology (SEQC) using a Roche Cobas Integra 400 plus analysers (Roche Diagnostics, Mannheim, Germany).

The Homeostasis Model Assessment index, used to estimate insulin resistance (HOMA-IR), was calculated using fasting blood glucose and insulin values [21]: [Fasting glucose (mg/dL) × Fasting insulin (mU/L)]/405.

The Homeostasis Model Assessment of β-Cell Function (HOMA-β) was calculated as follows [22]: [360 × Fasting Insulin (mU/L)]/[Fasting glucose (mg/dL) − 63].

The Quantitative Insulin Sensitivity Check Index (QUICKI) was also calculated using fasting insulin and glucose using this equation [23]: 1/[log fasting insulin (µIU/mL) − log fasting blood glucose (mg/dL)].

Systolic (SBP) and diastolic (DBP) blood pressure were measured using an automatic arm sphygmomanometer (OMRON, Healthcare M3; Model: HEM-7131-E; Shanghai, China). Measurements were carried out in both arms and then repeated two more times in the arm with higher blood pressure values following the protocol of the Spanish Society of Internal Medicine.

### 2.7. Statistical Analysis

Data were analysed using JASP (Version 0.18.1, Computer Software) and SPSS v22.0 software (SPSS Inc., Chicago, IL, USA). Descriptive data are presented in tables and expressed as the mean ± standard deviation, the coefficient of variation (CV) of the total study sample, expressed as a percentage, and the median with the interquartile range. CV values were used to observe inter-individual variability; if values were above 30%, data were considered heterogeneous [24]. The Shapiro–Wilk test was used to determine the variables that followed a normal distribution when data were segmented by sex (*n* < 50) and the Kolmogorov–Smirnov test was used to observe the distribution of the total study sample (*n* > 50). Data were analysed using Quantil–Quantil plots (Q-Q Plot) and histograms to confirm the assumption of normality. Levene’s test was used to contrast the homoscedasticity of variance between men and women in the analysed variables. Differences between men and women in continuous variables were analysed using Student’s *t*-test for independent groups if the assumption of normality and homoscedasticity were met; the Welch test was used if data followed a normal distribution but did not meet the assumption of homoscedasticity between variances; the Mann–Whitney *U* test was used if data did not follow a normal distribution. The Chi-Square test (*χ*^2^) was used to observe if there were differences in the distribution of men and women in some categorical variables of interest, such as the frequency of overweight or obesity and the frequency of metabolic syndrome. Correlations between quantitative continuous variables were analysed using the Pearson’s correlation coefficient (*r*) if the variables were normally distributed or Spearman’s correlation coefficient (*rho*) if not. *p*-values were considered significant if they were lower than 0.05 (α = 0.05).

To detect the common variation between original variables, several principal component analyses (PCA) were carried out: (1) with dietary intake variables to try to differentiate dietary patterns; (2) with biochemical, anthropometric, and body composition variables, and blood pressure measurements; (3) with variables of dietary intake, anthropometric, body composition, and biochemical analysis; (4) with variables of dietary intake, anthropometric, body composition, biochemical, blood pressure, and physical activity. Analyses 2, 3 and 4 were carried out to understand the relationship between the variables and see if the large number of variables could be simplified into a small number of factors. Variables that were not normally distributed were transformed using natural logarithms before introducing them in the PCA in order to achieve normality. A similar factor analysis approach has been followed in other works [25]. Variables were selected if there were sufficient significant correlations with coefficients above 0.30 but not too many correlations above 0.80 in the correlation matrix, since this could produce multi-collinearity problems. Moreover, variables with low communalities were excluded from factor analysis. As mentioned in the sample size paragraph, to assess the adequacy of the data, the Kaiser–Meyer–Olkin (KMO) test and the Bartlett test of sphericity were carried out. If KMO test indicated a value above 0.60 and the *p* value of the Bartlett test of sphericity was significant, data were considered adequate for the PCA. Factors were selected using Horn’s parallel analysis method with Varimax rotation. Once the main factors emerged, loadings above 0.40 were considered as relevant in the PCA.

## 3. Results

### 3.1. Subjects Sociodemographic Characteristics

The main sociodemographic characteristics of the sample population are shown in Table 1. The final sample constituted 84 volunteers (49 women and 35 men). All the participants lived in the urban area of Madrid and the surrounding areas. The overall sample population can be considered Europid in origin, since most participants were European. Only two volunteers were from Latin America countries, but they had been living in Spain for at least 6 years and had adopted the Spanish lifestyle, including Spanish dietary habits. Many participants came from the academic sector (graduate/postgraduate students, lecturers, researchers, and administrative personnel) and thus, according to the categories described by Stea et al. [26], most of them were included in the high education level category (73.8%), while only 17.5% had a medium educational level and 8.7% had a low education level (although all of them had at least secondary education). On average, the volunteers had a medium economic income, and no differences were found across the three educational levels (*p* = 0.229), indicating similar economic status.

The study population had a mean age of 49.7 ± 9.9 years, with a median age of 51.0 and an interquartile range (IQR) of 11.25 (56.00–44.75 for the third and first quartile, respectively). The distribution of age in the sample population is shown in Figure S1. About 86% of the population constituted adults over 40 years old (only 14% were under 40 years of age, between 20 and 38 years).

### 3.2. Dietary Intake

The results of the analysis of the dietary intake of the sample population are shown in Table 2 and Table 3. Initial dietary information could not be obtained from three volunteers (*n* = 81 for dietary intake). The mean energy density of the diet was 1.00 kcal/g, in line with other studies in adults with obesity [27]. Overall, the dietary intake of macronutrients was similar between men and women, except for carbohydrates, which was slightly but significantly higher in men (Table 2). Of note, and according to the Spanish Society of Community Nutrition (SENC) and the Nutritional Objectives (NO), the consumption of dietary fibre was below the recommendations (25 g/day for women and 35 g/day for men or 14 per 1000 kcal) and the dietary cholesterol and mg of cholesterol per 1000 kcal were above the recommended limit (<300 mg/day or <100 mg per 1000 kcal) [28].

When expressed as the percentage of energy coming from these macronutrients (Table 3), no differences were found between sexes, except for the percentage of energy coming from α-linolenic acid, which was higher in women (*p* = 0.029).

There was an imbalance in the energy profile of the diet as the percentages of energy intake from fat and protein exceeded the nutritional objectives for the Spanish population (30–35% of energy intake from fat and 10–15% from protein), at the expense of the energy intake from carbohydrates, which was lower than recommended (50–55%) (Table 3). However, the percentage of energy coming from alcohol was acceptable according to these nutritional objectives, as well as the intake of added sugar (nutritional objective for added sugars: ≤6% of total energy intake) [28].

The lipid profile of the diet was also unbalanced since the percentage of energy obtained from SFA and PUFA was above the recommended limit (NO: 7–8% and 5% of total energy intake, respectively), while the percentage from MUFA was below the recommendations. The energy intake from omega 3 fatty acids (W3), specially from α-linolenic acid, was lower than the limit settled by the SENC (NO: 1–2% of total energy intake); in contrast, linoleic acid and total omega 6 fatty acids (W6) consumption was higher than recommended (NO: 3% of total energy intake). Trans fatty acids intake was adequate (NO: <1% of total energy) (Table 3) [28].

Of special interest for this study is the variability of dietary data, expressed as CV%, with values ranging between approximately 25% and 40% for most of the examined dietary macronutrients (Table 2 and Table 3). The largest variability was found for added sugars (66.9%) and alcohol (>100%).

Regarding (poly)phenols, the estimated average total (poly)phenol intake was 1277.8 ± 808.7 mg/day (median: 1073.2 mg/day, IQR: 1017.2 mg/day) (Table 2). The highest total (poly)phenol intake that was recorded was 4582.6 mg/day and the lowest intake was 321.4 mg/day, with a large inter-individual variability also being displayed in the dietary daily intake of these compounds (CV = 63.3%). The main food groups and food items included in the estimation of the daily intake of (poly)phenols is shown in Supplementary Table S1. The distribution of (poly)phenol intake across the main analysed foods is shown in Supplementary Table S2. The most highly consumed (poly)phenol food sources (consumed by more than 75% of the participants) were cereals and derivatives, oils and olives, vegetables and fruits, processed foods, condiments, herbs, and tubers. The (poly)phenol food sources that were less consumed (by less than 40% of the participants) were soy and derivatives, juices, nuts, and legumes. Alcoholic drinks, cocoa and derivatives, and infusions were consumed by approximately half of the sample population. Of all the included food sources of (poly)phenols, the ones that most (>200 mg/day) contributed to the total daily (poly)phenol intake were legumes (504.5 ± 714.9 mg/day; 39.5%), cocoa and derivatives (371.0 ± 387.0 mg/day; 29.0%), fruits and derivatives (293.2 ± 207.0 mg/day; 23.0%), and nuts (216.6 ± 285.1 mg/day; 17.0%). The food sources that provided the lowest (<40 mg/day) content of (poly)phenols were herbs (2.8 ± 6.4; 0.2%), oils and olives (15.6 ± 11.5; 1.2%), condiments (21.6 ± 51.7 mg/day; 1.7%), and soy and derivatives (31.5 ± 36.4; 2.5%). The large CV (%) values (even >100%) indicate that the individuals’ consumption of (poly)phenols is very distant from the mean values and corroborate the large variability in the consumption of these bioactive compounds, as well as in the food sources of the compounds present in the diet of the participants.

### 3.3. Biochemical Measurements

Serum lipid profile, glucose metabolism measurements, hepatic enzymes, and high-sensitivity C-reactive protein (hsCRP) values are shown in Table 4. One blood sample could not be obtained from one volunteer due to difficulties in venipuncture (n = 83). Total cholesterol was higher than the reference limit settled by the Spanish Society of Clinical Biochemistry and Molecular Pathology (SEQC), which is 200 mg/dL. Other biochemical measurements, such as TG, LDL, VLDL, and HDL, were within the reference limits. HDL was significantly higher in women than in men (*p* < 0.001). Contrarily, median AST and ALT were found to be significantly higher in men compared to women (*p* = 0.014 and *p* = 0.006, respectively), but were within the reference limits in both sexes. hsCRP did not differ between sexes, and was also below the upper reference limit (Table 4).

Fasting blood glucose (FBG) and insulin were adequate according to the reference limits, but glycosylated haemoglobin (HbA1c) was higher than recommended (<5.7) in both sexes and could be indicative of predisposition to prediabetes, although the QUICKI values were comparable to those reported by Katz et al. [23] in obese subjects. HOMA-IR values were similar to those of healthy volunteers in other studies [29,30]. Regarding glucose metabolism, there were no significant differences between sexes in any of the measured parameters, as shown in Table 4. Mean systolic blood pressure (SBP) and diastolic blood pressure (DBP) were below 140/90 mmHg in both men and women, but men had higher values in both parameters and only SBP was significantly higher compared to women (*p* = 0.01).

Lower values of CV (≤30%) were found in the following biochemical and blood pressure measurements (Table 4): CT, HDL, LDL, HbA1c, FBG, QUICKI, SBP, and DBP. In contrast, TG, VLDL, insulin, HOMA-IR, HOMA-β, and ALT showed a CV of over 50%, higher than hsCRP (162.8%).

### 3.4. Anthropometric, Body Composition, Resting Metabolic Rate, and Physical Activity Measurements

Regarding body weight (Table 5), the mean BMI of the sample population was 30.5 ± 2.9 kg/m^2^. Among all the participants, 42.9% were overweight (n = 36), 50% had grade I obesity (n = 42), and 7.1% had grade II obesity (n = 6), with no significant differences in the distribution between men and women (data are included in Supplementary Table S3).

There were no significant differences between men (30.8 kg/m^2^) and women (30.3 kg/m^2^). These values exceed the recommended BMI settled by the WHO (18.5 kg/m^2^–24.9 kg/m^2^) and corroborate the higher associated risk for comorbidities in this population [31]. Moreover, mean waist circumference (WC) in both men and women was above the cut-off values defined by the International Diabetes Federation (≥94 cm for European men, and ≥80 cm for European women), showing a high prevalence of abdominal obesity in the study sample [32]. Despite having a similar BMI, waist circumference, hip circumference, the summation of the six skinfolds, and body composition measurements obtained by multifrequency BIA showed significant differences between women and men, as expected. The body composition analysis indicated that the body fat percentage and visceral fat area were significantly higher in women (*p* < 0.001 and *p* = 0.003, respectively), whilst percentage muscle mass (*p* < 0.001), skeletal muscle mass (SMM; *p* < 0.001), and skeletal muscle index (SMI; *p* < 0.001) were significantly lower compared to men (Table 5).

In this case, anthropometric and body composition measurements were more homogeneous in the study population, except for the visceral fat area (VFA), which exhibited CV values above the threshold of 30% (Table 5).

The results of a physical activity assessment, measured by accelerometers, are shown in Table 6, together with the RMR measured by calorimetry and data on energy expenditure. There are missing data from volunteers that failed to complete the physical activity or RMR measurements. The mean and median METs of the individuals were ≤1.5 METs, which indicates that participants had a sedentary lifestyle [33,34], in accordance with EFSA cut-off values for mean PAL. A prevalence of low physical activity among volunteers was also indicated, with no significant differences between sexes (Table 6). Average physical activity, energy expenditure, and METs did not differ between women and men, but women had a significantly higher step count per day than men (*p* = 0.048). Only two variables had a CV above the threshold of 30%, the average physical activity expenditure and the average of steps per day (Table 6), whereas the other variables had homogeneous inter-individual values.

To assess if the energy intake (EI) in the 24 h food recalls collected in the present work was underestimated, Goldberg and Black [35,36] equations were used to obtain the implementation of cut-off values to evaluate misreported EI. These equations consist of a calculation of the cut-off values, with specific intervals at a 95% confidence rate. To better understand the misreporting in the present study, the assessment was conducted at both group and individual levels using the calculated PAL values, RMR, as measured by calorimetry, and TEE. Afterwards, the EI:RMR ratio was calculated, considering that if the ratio was below the lower limit of the confidence interval (CI), participants underreported their EI, and if it was above the upper limit of the CI, participants overreported their EI [36]. Using this method at the individual level, 68.8% of the participants made an adequate report of their EI, 27.1% of the subjects underreported their EI, and 4.2% overreported their EI. Comparing the group of underreporters to those with an adequate report, although the EI (1477 ± 300 kcal/day and 2070 ± 351 kcal/day, respectively) and the energy density of the diet (0.875 ± 0.222 and 1.000 ± 0.179, respectively) was lower in underreporters, the BMI of both groups was similar (30.4 ± 1.7 kg/m^2^ and 30.8 ± 3.1 kg/m^2^, respectively).

### 3.5. Principal Component Analysis (PCA)

To explore the contribution to variability in the main factors associated with the overweight/obesity condition in the sample population, a PCA was carried out with different, sequential approaches. The variables included in the analysis are highly relevant, regarding diet composition, cardiometabolic health, body composition, and energy expenditure, and are widely accepted to be closely related to obesity. Some indexes were used, such as HOMA-IR or the waist/hip ratio; in these cases, the original variables were not included.

For the dietary factor analysis, energy intake, macronutrients, dietary cholesterol, dietary fibre, and (poly)phenols were included in the PCA. In this case, the KMO value was 0.787 and the *p* value of the Bartlett test of sphericity was significant (*p* < 0.001). These results show that the data were adequate for factor analysis. Once the PCA with the parallel analysis approach was conducted (*n* = 80), two main factors emerged, accounting for 64.5% of the total variance (Table 7). The first factor was responsible for 43.1% of the total variance and involved eight variables. It was mainly related to energy intake, lipids, and proteins; thus, it was called the “Energy intake factor”. The second factor was responsible for 21.4% of the total variance. It was composed of three variables and the higher positive loadings were total (poly)phenol intake, dietary fibre, and intrinsic sugars; thus, this factor could be closely related to fruits, vegetables, and legumes, which are food sources rich in these compounds, and could be named the “Plant-based diet factor”. Only the variable named added sugars showed high uniqueness, which means that it could not be included in these two factors. Complete data are shown in the Supplementary Material (Table S3).

The second PCA (*n* = 81) consisted of variables related to body composition, analysed through anthropometry and BIA, as well as variables related to cardiometabolic risk (biochemical measurements). The result of the KMO test was 0.670 and the *p* value of the Bartlett test was <0.001; therefore, the selected variables were adequate to perform a PCA. Three factors emerged, accounting for 73.2% of the total variance. The first factor accounted for 29.4% of the total variance and involved eight variables. This was called the “Cardiometabolic factor”, as it was positively loaded with TG, VLDL, the waist/hip ratio, HOMA-IR, SBP, DBP, and BMI, and negatively loaded with HDL. The second factor was related to body composition and anthropometric measurements; it was responsible for 27.5% of the total variance and included five variables. This factor was positively loaded with the percentage of body fat, the visceral fat area, the summation of six skinfolds, and BMI, and negatively loaded with the percentage of muscle mass. Therefore, it was called the “Adiposity factor”. Finally, the third factor was responsible for 16.2% of the total variance and was comprised of five variables. It was mainly loaded with total cholesterol and LDL, which is why it was called the “Serum cholesterol factor” (Table 8). Complete data are shown in the Supplementary Material (Table S4).

A third PCA was conducted using all the variables extracted in the other two PCAs (*n* = 77). The KMO value for this PCA was 0.677 and the *p* value of the Bartlett test was <0.001. Four factors arose, accounting for 65.4% of the total variance (Table 9). When the PCA with the parallel analysis was conducted, all the dietetic variables were associated with the first factor; thus, it was called the “Dietetic factor”. This factor, which involved twelve variables, was responsible for 24.0% of the total data variance. It was mainly related to energy intake, lipids, the lipid profile of the diet, and protein intake. Interestingly, the dietetic variables were not associated with the biochemical assessments or the body composition variables. The second factor was the “Cardiometabolic factor” (16.8% of the total variance) and comprised eight variables, the third factor was the “Adiposity factor” (15.2%) and included five variables, and the fourth factor was the “Serum cholesterol factor” (9.4%) and involved three variables. In this case, the second, third, and fourth factors revealed the same relationships between the variables as the second PCA that was conducted (Table 8). Complete data are shown in the Supplementary Material (Table S5).

A fourth PCA was carried out to observe the relationship between all these variables and the energy expenditure (TEE and METs); however, due to the smaller sample size (*n* = 48) and KMO value of 0.558, the data could not be analysed with a PCA approach.

## 4. Discussion

The current work aimed to contribute to our understanding of the variability and the relationship between some of the main factors that may explain overweight/obesity in a study population, before starting a dietary intervention consisting of the regular consumption of a lightly roasted coffee rich in (poly)phenols as a weight-loss/health improving tool. Therefore, we assessed the baseline characteristics of a study population with overweight and obesity, and then we aimed to identify the underlying patterns of data variation using a PCA. The novelty of this approach is that we selected many variables related to obesity and overweight and reduced the large dataset to simplify the interpretation of all the data collected from participants, unravelling associations that may not be clear when considering variables individually. Additionally, the generated components show the main sources of the data variability. By using this multivariate analysis, we obtained four factors, with the “dietetic factor” being the one responsible for the most data variability in this population with overweight and obesity, followed by the cardiometabolic factor and the adiposity factor, which agrees with the large variability found in the dietetic variables, followed by the biochemical variables, as well as in the visceral fat area.

It is well known that diet is a key factor in obesity prevention and treatment. As previously mentioned, current research addressing the factors causing inter-individual variability in response to dietary interventions incorporates rigorous analyses of dietary intake [10]. In the present work, 27.1% of participants underreported their EI, in line with previous studies that state that people with overweight or obesity tend to underreport their EI for different reasons, such as the following: underreporting food consumption deliberately, forgetting some food or beverages when they are interviewed (memory bias), or reducing and/or modifying their food consumption or even making different food choices due to the Hawthorne effect, consisting of participants changing their eating behaviour during the study period [37]. Therefore, a possible explanation for the high CV values observed in the dietetic variables (apart from the inter-individual differences naturally present in the volunteers) is the misreporting of the 24 h dietary recalls. Other dietary factors that have been extensively linked to obesity and overweight include excessive EI or elevated energy density in the diet, the high consumption of foods rich in SFA or added sugars [38,39,40,41], and unhealthy dietary patterns due to inadequate dietary choices. Accordingly, in the present study, participants’ diets were unbalanced, and their caloric and lipid profiles did not meet the nutritional objectives for the Spanish population [28].

Regarding sex differences in diet composition, only total carbohydrate intake was different between men and women (*p* = 0.038); however, when expressed as the energy coming from carbohydrates, it did not reach the threshold of significance. Nevertheless, women showed a lower carbohydrate intake trend (Table 2 and Table 3). These results dissent from previous studies showing that woman, regardless of their weight, consume more foods rich in carbohydrates and simple sugars, while men tend to have a higher consumption of food rich in fats [42]. In this work, total dietary fibre intake and dietary fibre corrected by energy (g/1000 kcal) were similar and lower than those recommended for both sexes, as well as (poly)phenol intake (total and corrected by energy). This indicates that the participants’ diets are low in fruits, vegetables, whole cereals, other sources of dietary fibre, and (poly)phenols. These results are uniform between both sexes (Table 2). Regarding sugar intake, intrinsic sugars and the percentage of energy coming from these sugars (Table 2 and Table 3) were similar to those described in the ANIBES study in a representative sample of the Spanish population between 18 and 64 years old [43].

At present, most authors agree that one dietary factor, or sole nutrients such as SFA or added sugars, should not be used to determine if a diet is healthy or not, as it is necessary to consider the dietary pattern as a whole to assess the influence of the diet on cardiometabolic or obesity risk [41]. In nutritional epidemiology, factor analysis is a commonly used method to determine eating patterns [25,44]. Therefore, in the present study, a factor analysis was used to reduce the dietary data collected with the 24 h recalls into patterns based upon the inter-correlations between nutrients and other bioactive compounds, such as fibre or (poly)phenol intake. Two factors emerged, named the “Energy intake factor” and the “Plant-based diet factor”, respectively, and the first was twice as responsible for the variance in the dietetic variables in this sample of people with overweight and obesity (43.1% and 21.4%, respectively; Table 7).

In addition to dietary intake, another key factor that must be considered to understand interindividual variability in weight loss is energy expenditure, which includes physical activity and resting energy requirements [10]. In fact, lifestyle changes to balance EI and energy expenditure are more realistic strategies to treat an excess of body fat [45]. Regarding physical activity in the present study population, the median MET values indicate that most volunteers had a sedentary behaviour (median MET values 1.12, Table 6). This outcome was reinforced by individual PAL values (calculated as the TEE:RMR ratio), which indicated that most participants had low levels of physical activity (mean PAL value 1.36) and only one subject had moderate physical activity levels (PAL value above 1.6), according to the EFSA cut-off values [20,36]. In agreement with this, in a recent meta-analysis, an increased risk of obesity was correlated with sedentary behaviour and physical inactivity [34].

Sex-specific differences regarding body composition, anthropometry, biochemical measurements, and energy expenditure are widely documented (Table 5). Interestingly, in the present study, female subjects had a significantly higher VFA than men (*p* = 0.003), indicating a central distribution of adiposity. This elevated accumulation of fat in visceral tissues could be explained by the age of the female subjects (median age 53 years), as visceral fat accumulation tends to increase due to menopause and, in postmenopausal women, visceral fat represents nearly 15–20% of total fat [46]. Although this excess of visceral fat can increase the cardiometabolic risk in postmenopausal women [46], women did not exhibit a higher metabolic risk compared to men when the serum lipid profile, glucose metabolism, or other measurements were analysed (Table 4). In fact, women were observed to have higher HDL levels compared to men, related to differences in sex hormones, mainly due to oestrogens [47] and lower levels of transaminases (AST and ALT) and SBP (Table 4). Other studies suggest that liver transaminases levels are associated with sex, since men usually have higher levels of ALT [48,49,50,51]. In the present work, although men had higher transaminases levels, both sexes had values within the reference limits (Table 4). SBP was also higher in men than in women, in accordance with previous research suggesting that the higher prevalence of hypertension in men may be due to interactions between oestrogens/testosterone and the renin–angiotensin–aldosterone system. Nonetheless, women appeared to have a higher cardiovascular risk at lower blood pressure levels than men, and once menopause is reached, blood pressure increases at higher rates in women [52,53].

When tackling overweight and obesity using a personalized approach, many factors may be considered [17]; thus, it is important to understand the relationship between them and it may be convenient to transform a large set of correlated variables into smaller sets of non-correlated variables, called principal components or factors [25,44]. In the present work, this approach was carried out using dietary items, total energy expenditure, body composition, and biochemical and blood pressure baseline biomarkers. However, as previously mentioned, the PCA presented certain limitations, as the KMO value was under 0.6 (0.565). Moreover, in this analysis, we used a smaller sample size because of the variables related to energy expenditure (TEE, METs, and average steps per day), as explained in the Limitations section. Furthermore, we observed that the energy expenditure factor contributed little (8.7%) to the total variability. Therefore, this factor was removed to improve the analysis of the study population.

After removing the energy expenditure factor, twenty-six variables were reduced into four factors, explaining 65.4% of the total variance (Table 9). The first principal component in our study, accounting for 24% of the total data variability, was composed of dietary variables including total lipid intake, lipid composition (MUFA, SFA, PUFA), proteins, and dietary cholesterol (the “Dietetic factor”; Table 9), in line with that reported by Garaulet et al. [54], who also used the PCA approach, along with dietary, body composition, and biochemical variables. These results point to energy intake, energy-dense foods, animal products, and foods with a high lipid content as being responsible for most of the variability in the dietetic factor. Moreover, this result agrees with the dietary unbalance identified using the 24 h recall dietary analysis, which showed high intake of lipids, proteins, and cholesterol (Table 2 and Table 3). However, with the dietary analysis software used (DIAL), the source of protein (i.e., animal or plant) cannot be distinguished. Considering that dietary cholesterol is only found in animal products, and due to the strong correlation found between protein intake and dietary cholesterol, both corrected by EI (*rho* = 0.509, *p* < 0.001), it could be inferred that the main source of protein in the participants’ diet was of animal origin. The high consumption of protein coming from plant-based foods (legumes, nuts, seeds) has been associated with better health outcomes [55], whilst a higher consumption of animal products, specifically red or processed meat, has been related to cardiovascular diseases, mainly due to their high content of SFA [56]. As previously mentioned, the subjects of this study exceeded the recommended intake of SFA and protein, while their dietary fibre intake was low, as was the “Plant-based diet factor” (21.4%) (Table 7), corroborating the high consumption of animal-based products (Table 3).

(Poly)phenol intake values in the present work (1073 mg/day, Table 1) were similar to those described in the EPIC study in Mediterranean countries (1011 mg/day) [57]. In previous studies, these compounds showed anti-obesity properties that could be explained by different mechanisms, such as modulating neuro-hormones that play a role in satiety, like neuropeptide Y, supressing lipogenesis, or even modifying the microbiota. Therefore, the modification of dietary habits to increase the consumption of plant-based foods rich in phenolic compounds, including the consumption of a (poly)phenol-rich coffee, could help to reduce the risk of developing obesity and/or treat comorbidities related to features of obesity/metabolic syndrome in certain subjects depending on the baseline features [58,59]. This idea is based on a previous human study in which a blend of green/roasted coffee rich in (poly)phenols, was regularly consumed at a realistic dose (3 cups/day) by healthy and hypercholesterolemic subjects. The subjects with a higher cardiovascular risk showed an improvement in cardiometabolic risk markers and abdominal adiposity. Another important outcome of that study was the high inter-individual variability in the analysed biomarkers [60,61,62]. Not only did the percentage of responders and non-responders vary between the two study groups, but there was also a large variability in the extent of the responses, with variations in the range of 1–115 units in TC or TG.

Even though the “Dietetic factor” (EI, lipids, and proteins) explained most of the variability (24%), the “Cardiometabolic factor” and “Adiposity factor” had a considerable and quite similar contribution to total variability in the PCA (16.8% and 15.2%, respectively; Table 9). The relevance of the secondary factor related to adiposity (% body fat, visceral fat area, etc.) may be attributed to the adipose tissue not only serving as an energy reservoir but also as a secretory organ of certain molecules that have endocrine, paracrine, and autocrine actions, which are involved in the regulation of body weight (leptin, adiponectin), in the local inflammation generated in obesity (tumour necrosis factor α, interleukin (IL)-6, and IL-1β), or in vascular function (angiotensin II and plasminogen activator inhibitor-1) [63,64]. The low-grade inflammation in obesity is closely related to insulin resistance and angiogenesis, which would explain the importance of the “Cardiovascular risk factor” (main loadings: TG, VLDL, waist/hip ratio, and HDL). Accordingly, most participants had elevated abdominal and subcutaneous fat, as measured by different indices (Table 5), and a high cardiometabolic risk due to their total cholesterol and glycosylated haemoglobin (HbA1c) values (Table 4).

In sum, an unbalanced ratio between energy intake and expenditure, along with an inadequate diet, might trigger body composition changes by elevating the levels of subcutaneous and visceral fat. Due to the existence of different obesity phenotypes, some people can accumulate fat more easily in the visceral adipose tissue, while others have greater accumulation in subcutaneous adipose tissue. In this study population, although most body composition parameters were homogeneous, a great variability was found in the visceral fat, which could explain the presence of different obesity phenotypes in this population and the great variability found in the biochemical variables. These changes in the visceral adiposity may alter the serum lipid profile, leading to hypercholesterolemia, hypertriglyceridemia, insulin resistance, and inflammation, thus connecting obesity, particularly abdominal obesity, with metabolic syndrome, type 2 diabetes mellitus, and cardiovascular disease [64].

### Strengths and Limitations

A strength of this study is that participants were exhaustively characterised using objective and accurate techniques: the measurement of physical activity was taken with accelerometers, the assessment of RMR was made using indirect calorimetry, and the diet was systematically measured using three 24 h dietary recalls, which enabled the meticulous estimation of (poly)phenol intake. Body composition was rigorously measured by anthropometry and BIA. However, there are some limitations in the present work. The sample size (*n* = 84) was large enough to carry out a crossover intervention to study the effects of the sustained intake of a (poly)phenol-rich coffee on overweight/obesity, including the variability in the responses, although a higher number of participants would have been preferred in the multivariate analyses. In addition, some of the data from accelerometers were missing due to attrition bias; it seems that some volunteers did not wear the accelerometer for the indicated time or the equipment did not register the data properly.

Therefore, due to missing data, it was not possible to carry out a final PCA to observe the relationship between energy expenditure, diet, body composition, and biochemical measurements. Furthermore, as these data were necessary to estimate the misreporting of the EI, it was not feasible to perform a sub-analysis based on subgroups of people that underreported, overreported, or adequately reported their EI. Since eliminating those who underreported or overreported their EI might bias the data, the PCA was performed using all participants, without segmenting. It could be hypothesized that the high CVs of the dietetic variables were because of the misreporting in some individuals; however, due to the lower sample size (*n* = 13 underreported, *n* = 33 had an adequate report, *n* = 2 overreported), the analysis could not be carried out. Moreover, the CI of the cut-off values at the individual level (*n* = 1) was wider and could be limited in its ability to detect invalid reports. However, some limitations of these methods were avoided by calculating PAL at the individual level, using more reliable tools to measure physical activity (by accelerometers) and RMR (by calorimetry). This approach is more accurate in identifying the misreporting of dietary recalls at the individual level [36].

To end, due to selection bias and the aforementioned limitations, the results cannot be applied to the general population with overweight or obesity, but the present work will help to understand the response of the subjects involved in the GREENCOF nutritional intervention. We acknowledge that not all the factors related to obesity were included in the present analysis due to its complexity and extension. Factors like sex, age, ethnicity, socioeconomic status, and education, which were described in the characterization of the sample population, were not included in the present analysis due to the relative homogeneity of these variables in the sample population. Other important factors, such as genetics, the intestinal microbiota, and the circadian rhythmicity, will be addressed in future communications of the GREENCOF project.

## 5. Conclusions

In the present sample population of men and women with overweight or obesity, large variability was found in the intake of specific dietary items, biochemical variables, and physical activity measurements. In contrast, anthropometric and body composition measurements were more homogenous, except for the visceral fat area. When all the variables related to overweight and obesity were analysed using PCA, diet was the principal factor responsible for most of the variability in the data of people with overweight and obesity, followed by the cardiometabolic factor and the adiposity factor. The latter outcome may be attributed to the important role of the adipose tissue as a secretory organ of molecules involved in the regulation of body weight, inflammation, and vascular function.

## Figures and Tables

**Table 1:** Sociodemographic characteristics of the study participants (based on self-reported questionnaires).

	Absolute Frequency (*n*)	Relative Frequency (%)
Men	35	41.7
Women	49	58.3
European origin	78	97.5
Latin-American origin	2	2.5
Low educational level	7	8.7
Medium educational level	14	17.5
High educational level	59	73.8
	Median (IQR)	
Monthly income per person (€)	1167 (900)	
Monthly income per family unit (€)	3000 (1900)	
Age	51.0 (11.3)	

Low educational level: no education, or primary or secondary education. Medium educational level: upper secondary education. High educational level: advanced vocational education or university degree. There are some missing data since some participants did not provide an answer to questions relative to education, income, or origin.

**Table 2:** Daily edible food intake, energy intake, energy density, macronutrients, alcohol, dietary fibre, and total (poly)phenols intake recorded in three dietary recalls of 24 h.

	Total (*n* = 81)		Men (*n* = 35)		Women (*n* = 46)		*p* Value
	Mean ± SD (%CV)	Median (IQR)	Mean ± SD	Median (IQR)	Mean ± SD	Median (IQR)	
Edible food intake (g) ^a^	2091 ± 572 (27.3%)	2038 (646)		2053 (513)		1967 (773)	0.498
Energy intake (kcal)	2015 ± 521 (25.9%)	1960 (716)	2095 ± 509		1953 ± 527		0.227
Energy density (kcal/g) ^b^	1.00 ± 0.25 (24.8%)	1.00 (0.32)	1.01 ± 0.20		0.99 ± 0.28		0.643
Proteins (g) ^a^	88.9 ± 23.9 (26.9%)	88.8 (22.4)		88.8 (18.6)		87.2 (28.4)	0.985
Carbohydrates (g)	186 ± 56 (30.1%)	179 (72)	200.6 ± 57.6		174.7 ± 52.6		0.038 *
Simple sugars (g) ^a^	75.8 ± 27.7 (36.6%)	70.8 (34.2)		73.1 (29.3)		68.1 (37.1)	0.448
Intrinsic sugars (g)	47.9 ± 17.6 (36.8%)	48.3 (22.5)	47.9 ± 17.7		47.9 ± 17.8		0.986
Added sugars (g) ^a^	28.1 ± 18.8 (66.9%)	24.9 (20.3)		25.8 (20.1)		23.5 (21.2)	0.381
Lipids (g) ^a^	92.1 ± 29.9 (32.4%)	86.8 (42.5)		90.4 (46.4)		83.7 (44.1)	0.706
SFA (g) ^a^	28.6 ± 10.7 (37.5%)	24.6 (17.0)		25.3 (16.2)		24.3 (17.2)	0.706
MUFA (g) ^a^	40.5 ± 14.3 (35.3%)	37.5 (13.7)		37.3 (13.1)		38.7 (15.3)	0.838
PUFA (g) ^a^	12.3 ± 5.3 (43.2%)	11.2 (5.3)		11.2 (5.4)		11.3 (4.6)	0.637
W6 (g) ^a^	10.3 ± 4.9 (47.6%)	9.2 (5.0)		9.7 (5.5)		8.8 (4.9)	0.520
W3 (g) ^a^	1.9 ± 1.0 (50.6%)	1.5 (1.1)		1.40 (0.95)		1.80 (1.15)	0.359
W6/W3 ratio ^a^	6.5 ± 3.8 (59.0%)	6.1 (4.5)		6.43 (3.98)		5.05 (4.67)	0.564
Total dietary cholesterol (mg) ^a^	339 ± 132 (39.0%)	317 (163)		317 (157)		319 (161)	0.577
Cholesterol (mg/1000 kcal) ^a^	172.3 ± 68.5 (39.7%)	166.1 (80.6)		166.1 (69.1)		169.1 (90.5)	0.659
Alcohol (g) ^a^	6.7 ± 9.6 (143.9%)	2.9 (10.1)		4.1 (13.3)		2.8 (7.9)	0.503
Dietary fibre (g) ^a^	19.8 ± 8.1 (40.7%)	19.7 (10.5)		19.8 (11.8)		19.2 (8.4)	0.501
Dietary fibre (g/1000 kcal) ^a^	10.0 ± 3.5 (34.7%)	9.7 (5.0)		9.2 (3.9)		10.1 (5.0)	0.370
Total (poly)phenols (mg) ^a^	1278 ± 809 (63.3%)	1073 (1017)		1073 (688)		1075 (1095)	0.802
(Poly)phenols (mg/1000 kcal) ^a^	642 ± 371 (57.8%)	535 (426)		509 (412)		594 (437)	0.659

Energy intake was expressed as kcal per day; macronutrients, dietary fibre, and alcohol intake was expressed as grams per day and cholesterol as mg per day. Dietary fibre and cholesterol intake were also expressed in grams or milligrams per 1000 kcal, calculated as follows: [(dietary cholesterol (mg) or dietary fibre (g))/energy intake (kcal)] × 1000. Data are presented as mean ± standard deviation (SD), with median and interquartile range (IQR) in parentheses. Segmented data are only presented as mean or median, depending on normality. The superscript a indicates data that do not follow a normal distribution when data are segmented, and b indicates heteroscedasticity between variances of men and women. Significant *p*-values are marked with one asterisk (*) for *p* < 0.05. SFA: saturated fatty acids; MUFA: monounsaturated fatty acids; PUFA: polyunsaturated fatty acids; W6: Omega 6 fatty acids; W3: Omega 3 fatty acids.

**Table 3:** Percentages of daily energy intake from macronutrients, alcohol, and dietary fibre.

	Total (*n* = 81)		Men (*n* = 35)		Women (*n* = 46)		*p* Value
	Mean ± SD (%CV)	Median (IQR)	Mean ± SD	Median (IQR)	Mean ± SD	Median (IQR)	
% Carbohydrates	36.9 ± 6.4 (17.4%)	37.1 (10.3)	38.4 ± 6.6		35.8 ± 6.1		0.067
% Simple sugars	14.9 ± 4.1 (27.4%)	14.7 (4.9)	15.1 ± 4.7		14.8 ± 3.6		0.792
% Intrinsic sugars	9.7 ± 3.4 (35.1%)	9.1 (4.9)	9.3 ± 3.5		10.0 ± 3.3		0.352
% Added sugars	5.4 ± 3.1 (57.0%)	5.1 (4.2)	5.6 ± 3.4		5.2 ± 2.8		0.541
% Proteins	17.9 ± 2.9 (16.3%)	17.8 (4.1)	17.2 ± 2.8		18.3 ± 2.9		0.089
% Lipids	40.9 ± 6.0 (14.6%)	40.7 (9.7)	39.5 ± 5.8		41.9 ± 6.0		0.072
% SFA ^a^	12.6 ± 2.6 (20.7%)	12.4 (3.7)		12.0 (3.5)		13.1 (3.5)	0.113
% MUFA	18.1 ± 3.8 (21.2%)	17.5 (4.5)	17.2 ± 3.5		18.7 ± 4.0		0.096
% PUFA ^a^	5.5 ± 1.8 (33.2%)	5.2 (2.2)		5.1 (2.6)		5.3 (1.9)	0.396
% W3 ^a^	0.87 ± 0.50 (52.3%)	0.76 (0.59)		0.61 (0.44)		0.87 (0.55)	0.088
% α-Linolenic acid ^a^	0.5 ± 0.2 (42.3%)	0.44 (0.23)		0.40 (0.17)		0.49 (0.22)	0.029 *
% W6 ^a^	4.6 ± 1.7 (37.0%)	4.1 (2.1)		4.3 (2.3)		4.1 (1.9)	0.652
% Linoleic acid ^a^	4.5 ± 1.7 (37.5%)	4.1 (2.1)		4.1 (2.2)		4.0 (1.8)	0.744
% Trans FA	0.38 ± 0.19 (49.3%)	0.38 (0.27)	0.38 ± 0.17	0.36 (0.23)	0.39 ± 0.21	0.41 (0.29)	0.871
% Alcohol ^a^	2.3 ± 3.3 (145.5%)	1.0 (3.2)		1.2 (4.3)		1.0 (2.5)	0.565
% Dietary fibre ^a^	2.0 ± 0.7 (34.7%)	1.9 (1.0)		1.8 (0.8)		2.0 (1.0)	0.370

Results are expressed as the percentage of total energy intake from macronutrients, alcohol, and dietary fibre, considering that protein and carbohydrates provide 4 kcal per gram, lipids provide 9 kcal per gram, alcohol provides 7 kcal per gram, and dietary fibre provides 2 kcal per gram. Data are presented as mean ± standard deviation (SD), with median and interquartile range (IQR) in parentheses. Segmented data are only presented as mean or median, depending on normality. The superscript a indicates data that do not follow a normal distribution when data are segmented by sex. Significant *p*-values are marked with one asterisk (*) for *p* < 0.05. FA: fatty acids; SFA: saturated fatty acids; MUFA: monounsaturated fatty acids; PUFA: polyunsaturated fatty acids; W6: Omega 6 fatty acids; W3: Omega 3 fatty acids.

**Table 4:** Serum lipid profile, glucose metabolism measurements, hepatic enzymes, CRP, and blood pressure.

	Total (*n* = 83)		Men (*n* = 35)		Women (*n* = 48)		
	Mean ± SD (%CV)	Median (IQR)	Mean ± SD	Median (IQR)	Mean ± SD	Median (IQR)	*p* Value
TC (mg/dL)	211.0 ± 31.1 (14.7%)	214 (42.5)	204.1 ± 31.7		216.0 ± 30.2		0.085
TG (mg/dL) ^a^	127.7 ± 68.1 (53.3%)	111 (52)		121 (61.5)		104.5 (50)	0.091
HDL (mg/dL) ^a^	61.6 ± 17.0 (27.6%)	56 (26)		51 (14)		68 (23.3)	<0.001 ***
LDL (mg/dL)	124.0 ± 25.0 (20.2%)	126.8 (36.6)	120.9 ± 25.6		126.2 ± 24.6		0.337
VLDL (mg/dL) ^a^	25.5 ± 13.6 (53.2%)	22.0 (10.3)		24.0 (12.0)		21.0 (10.3)	0.094
HbA1c (%)	5.75 ± 0.35 (6.1%)	5.70 (0.40)	5.71 ± 0.32		5.77 ± 0.37		0.504
FBG (mg/dL)	93.1 ± 11.3 (12.2%)	92 (12.5)	92.3 ± 12.0		93.7 ± 11.0		0.586
Insulin (µUI/mL) ^a^	10.5 ± 5.7 (54.2%)	9.2 (6.6)		10.5 (5.1)		8.0 (7.0)	0.173
HOMA-IR ^a^	2.41 ± 1.32 (54.5%)	1.98 (1.66)		2.41 (1.22)		1.84 (2.05)	0.243
HOMA-β ^a^	146 ± 123 (84.2%)	111 (97)		119 (123)		98 (83)	0.437
QUICKI	0.343 ± 0.027 (7.8%)	0.344 (0.040)	0.340 ± 0.027		0.345 ± 0.026		0.417
AST (UI/L) ^a^	24.8 ± 12.0 (48.4%)	22.0 (8.0)		24.0 (9.3)		21.0 (5.5)	0.014 *
ALT (UI/L) ^a^	30.1 ± 18.9 (62.9%)	24.0 (16.0)		30.0 (20.0)		21.0 (10.0)	0.006 **
hsCRP (mg/dL) ^a^	0.345 ± 0.562 (162.8%)	0.153 (0.301)		0.158 (0.248)		0.151 (0.440)	0.843
Blood pressure							
SBP (mmHg) ^a^	126.2 ± 18.6 (14.7%)	124.7 (25.1)		130.7 (18.7)		121.3 (25.0)	0.01 *
DBP (mmHg) ^a^	84.8 ± 10.9 (12.8%)	82.0 (14.8)		87.3 (18.6)		80.7 (12.7)	0.110

Data are presented as mean ± standard deviation (SD), with median and interquartile range (IQR) in parentheses. The superscript a indicates data that do not follow a normal distribution when data are segmented by sex. Segmented data are only presented as mean or median, depending on normality. Significative *p*-values are marked with one asterisk (*) for *p* < 0.05, two asterisks (**) for *p* < 0.01, or three asterisks (***) for *p* < 0.001. TC: total cholesterol; TG: triglycerides; HDL: high-density lipoprotein; LDL: low-density lipoprotein; VLDL: very low-density lipoprotein. HbA1c: glycated haemoglobin; FBG: fasting blood glucose; HOMA-IR: Homeostasis Model Assessment of insulin resistance; HOMA-β: Homeostasis Model Assessment of β-cell function; QUICKI: Quantitative Insulin Sensitivity Check Index. AST: aspartate aminotransferase; ALT: alanine aminotransferase; hsCRP: high-sensitivity C-reactive protein. SBP: systolic blood pressure; DBP: diastolic blood pressure.

**Table 5:** Anthropometric and body composition measurements.

	Total (*n* = 84)		Men (*n* = 35)		Women (*n* = 49)		
	Mean ± SD (%CV)	Median (IQR)	Mean ± SD	Median (IQR)	Mean ± SD	Median (IQR)	*p* Value
Height (cm)	165.4 ± 8.1 (4.9%)	163.6 (13)	172.1 ± 6.7		160.7 ± 5.0		<0.001 ***
Weight (kg) ^b^	83.6 ± 11.1 (13.2%)	83.6 (12.0)	91.2 ± 10.9		78.1 ± 7.4		<0.001 ***
BMI (kg/m^2^)	30.5 ± 2.9 (9.6%)	30.4 (3.5)	30.8 ± 2.8		30.3 ± 3.0		0.471
WC (cm)	96.0 ± 11.5 (11.9%)	94.7 (15.4)	102.2 ± 11.1		91.5 ± 9.5		<0.001 ***
HC (cm) ^a^	108.5 ± 6.8 (6.3%)	106.8 (10.4)		104.5 (6.2)		110.2 (10)	<0.001 ***
WC/HC ^b^	0.89 ± 0.11 (12.8%)	0.88 (0.15)	0.97 ± 0.11		0.83 ± 0.08		<0.001 ***
WC/Height	0.58 ± 0.07 (11.3%)	0.58 (0.09)	0.59 ± 0.06		0.57 ± 0.07		0.107
SUMM 6 FOLDS ^a^	153.6 (33.0) (21.5%)	162.1 (46.5)		130.0 (36.8)		170.2 (24.6)	<0.001 ***
Body composition measured by bioimpedance							
Fat weight (kg)	30.2 ± 7.3 (24.0%)	29.8 (9.7)	28.4 ± 7.6		31.5 ± 6.8		0.053
% Body fat	36.2 ± 7.8 (21.5%)	35.6 (11.8)	30.8 ± 6.5		40.1 ± 6.2		<0.001 ***
VFA (cm^2^) ^a^	141.9 ± 43.6 (30.7%)	137.6 (70.7)		115.4 (53.2)		155.9 (56.2)	0.003 **
SMM (kg) ^b^	29.9 ± 6.4 (21.5%)	27.7 (9.3)	35.9 ± 5.2		25.7 ± 2.9		<0.001 ***
% Muscle mass	35.7 ± 5.1 (14.3%)	35.8 (7.0)	39.4 ± 4.5		33.1 ± 3.6		<0.001 ***
SMI (kg/m^2^) ^b^	7.94 ± 0.95 (12.0%)	7.70 (1.40)	8.79 ± 0.74		7.33 ± 0.51		<0.001 ***

Data are presented as mean ± standard deviation (SD), with median and interquartile range (IQR) in parentheses. Segmented data are only presented as mean or median, depending on normality. Superscript a indicates data that do not follow a normal distribution when data are segmented; the letter b indicates heteroscedasticity between the variance of men and women. Significant *p*-values were marked two asterisks (**) for *p* < 0.01, or three asterisks (***) for *p* < 0.001. BMI: body mass index; WC: waist circumference; HC: hip circumference; SUMM 6 FOLDS: summation of six skinfolds (tricipital, bicipital, subscapular, suprailiac, mid-thigh, and calf); VFA: visceral fat area; SMM: skeletal muscle mass; SMI: skeletal muscle index.

**Table 6:** RMR and physical activity measurements.

	Total		Men		Women		
	Mean ± SD (%CV)	Median (IQR)	Mean± SD	Median (IQR)	Mean± SD	Median (IQR)	*p* Value
RMR (kcal/day) (*n* = 56)	1778 ± 310 (17.4%)	1712 (472)	2038 ± 240		1596 ± 206		<0.001 ***
TEE (*n* = 50)	2427 ± 453 (18.7%)	2372 (507)	2723 ± 419		2194 ± 329		<0.001 ***
Average PAE (kcal/day) ^a^ (*n* = 60)	463 ± 185 (39.9%)	447(169.1)		443.5 (190.7)		454.1 (140.6)	0.662
METs ^a^ (*n* = 60)	1.14 ± 0.09 (7.6%)	1.12 (0.09)		1.12 (0.10)		1.12 (0.08)	0.676
Steps per day ^a^ (*n* = 60)	7961 ± 3149 (42.0%)	7281 (3445)		6866 (2048)		8193 (3353)	0.048 *
PAL (*n* = 50)	1.36 ± 0.10 (7.0%)	1.36 (0.11)	1.34 ± 0.10		1.38 ± 0.09		0.084

Data are presented as mean ± standard deviation (SD), with median and interquartile range (IQR) in parentheses. Segmented data are only presented as mean or median, depending on normality. The letter a indicates data that do not follow a normal distribution when data are segmented by sex. Significant *p*-values are marked with one asterisk (*) for *p* < 0.05 or three asterisks (***) for *p* < 0.001. RMR: resting metabolic rate; TEE: total energy expenditure; average PAE: physical activity expenditure; MET: metabolic equivalent of task. 1 MET = 3.5 millilitres of oxygen per kilogram of body weight per minute (mL/kg/min). PAL: physical activity level.

**Table 7:** Component-rotated array from the PCA of the diet, eigenvalues, and percentage of total variance of each factor.

	PC1	PC2	Uniqueness
Energy Intake	0.903		0.062
Lipids	0.896		0.120
SFA ^a^	0.895		0.180
Proteins ^a^	0.830		0.287
MUFA ^a^	0.801		0.279
Dietary cholesterol	0.634		0.591
Carbohydrates	0.623		0.515
PUFA ^a^	0.615		0.510
(Poly)phenol ^a^		0.881	0.213
Dietary fibre ^a^		0.814	0.274
Intrinsic sugars		0.762	0.393
Added sugars ^a^			0.836
Eigenvalues	6.123	1.617	
Percentage of total variance	43.1	21.4	

Extraction method: principal component analysis using parallel analysis. Rotation method: Varimax. Superscript a indicates variables that were transformed using natural logarithms (Ln) to achieve a normal distribution before introducing them into the PCA. Loadings under 0.4 were not registered.

**Table 8:** Component-rotated array from the PCA of anthropometric, body composition, and biochemical measurements, eigenvalues, and the percentage of total variance of each factor.

	PC1	PC2	PC3	Uniqueness
TG ^a^	0.860			0.259
VLDL ^a^	0.857			0.264
Waist/hip ratio	0.764			0.379
HDL ^a^	−0.731		0.471	0.238
HOMA-IR ^a^	0.684			0.457
SBP	0.609		0.427	0.447
DBP	0.560		0.429	0.502
BMI	0.535	0.643		0.279
% Body fat		0.972		0.042
% Muscle mass		−0.941		0.086
Visceral fat area		0.932		0.086
SUMM 6 skinfolds ^a^		0.812		0.286
Total cholesterol			0.914	0.159
LDL			0.853	0.270
Eigenvalues	4.268	3.717	2.260	
Percentage of total variance (rotated solution)	29.4	27.5	16.2	

Extraction method: principal component analysis using parallel analysis. Rotation method: Varimax. Letter a indicates variables that were transformed using natural logarithms (Ln) in order to achieve a normal distribution before introducing them into the PCA. Loadings under 0.4 were not registered.

**Table 9:** Component-rotated array from the PCA of diet, anthropometric, body composition and biochemical measurements, eigenvalues, and percentage of total variance of each factor.

	PC1	PC2	PC3	PC4	Uniqueness
Energy Intake	0.950				0.072
Lipids	0.921				0.145
SFA ^a^	0.865				0.239
MUFA ^a^	0.839				0.275
Proteins ^a^	0.792				0.254
PUFA ^a^	0.702				0.492
Carbohydrates	0.678				0.457
Dietary fibre	0.570				0.545
Dietary cholesterol	0.523				0.567
Intrinsic sugars	0.510				0.731
Polyphenols ^a^	0.487				0.716
Added sugars ^a^	0.411				0.711
TG ^a^		0.871			0.220
VLDL ^a^		0.868			0.224
Waist/hip ratio		0.764			0.386
HDL ^a^		−0.726		0.472	0.241
HOMA-IR ^a^		0.683			0.477
SBP		0.589			0.536
DBP ^a^		0.572			0.529
BMI		0.519	0.653		0.269
% Body fat			0.960		0.047
% Muscle mass			−0.926		0.090
Visceral fat area			0.923		0.087
SUMM 6 skinfolds			0.790		0.321
Total cholesterol				0.928	0.129
LDL				0.868	0.243
Eigenvalues	6.734	4.394	3.431	2.440	
Percentage of total variance (rotated solution)	24.0	16.8	15.2	9.4	

Extraction method: principal component analysis. Rotation method: Varimax with Kaiser normalization. The rotation converged in seven iterations. Letter a indicates variables that were transformed using natural logarithms (Ln) in order to achieve a normal distribution before introducing them into the PCA. Loadings under 0.5 were not registered.

## Data Availability

Data is contained within the article (and Supplementary Materials).

## Supplementary Materials

The following supporting information can be downloaded at: https://www.mdpi.com/article/10.3390/nu16081143/s1.
