# Supplementary material for: Characterizing Factors Associated with Excess Body Weight: A Descriptive Study Using Principal Component Analysis in a Population with Overweight and Obesity
Source: Nutrients. 2024 Apr 12;16(8):1143. doi: 10.3390/nu16081143 (PMC11054611; doi:10.3390/nu16081143)
Supplement: Supplementary file 1 [file nutrients-16-01143-s001.zip › nutrients-2869238-supplementary.pdf]

nutrients

Supplementary information

Characterizing Factors Associated with Excess Body Weight: A Descriptive Study Using Principal Component Analysis in a Population with Overweight and Obesity

Álvaro Fernández-Cardero [1], José Luis Sierra-Cinos [2,3], Adrián López-Jiménez [1], Beatriz Beltrán [2], Carmen Cuadrado [2], María Teresa García-Conesa [4], Laura Bravo [1] and Beatriz Sarriá [1,2,*]

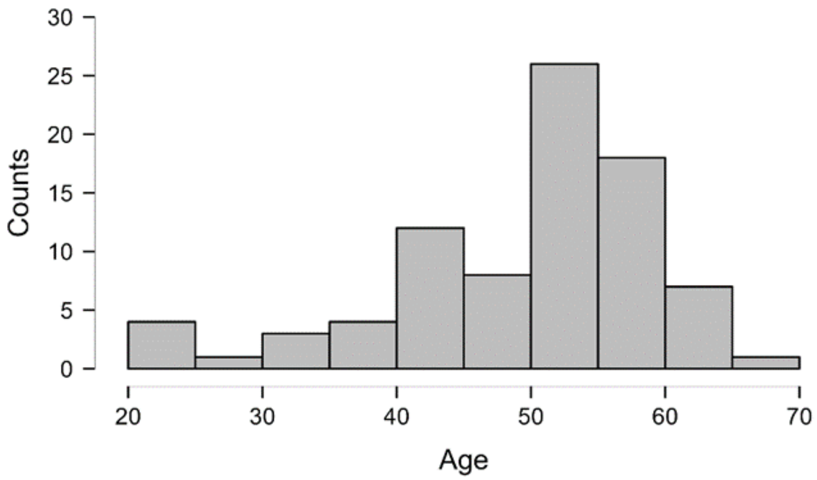

Figure S1. Histogram of subjects' age.

Table S1. Main food groups and food items included in the estimation of the daily dietary polyphenol intake of the overweight/obese participants. Based on the average results of the food items consumed by the participants (as analysed by the DIAL software for assessing diets and food calculations) coupled with the content of total (poly)phenols in the Phenol Explorer database.

Food group	Main food items included
**Cereals and derivatives**	Bread (white, grain, rye), breakfast cereals (bran, muesli, oat), wheat flour (refined, whole grain), pasta (normal, whole grain), rice (boiled), oat, maize.
**Oils and olives**	Olive oil (refined, virgin, extra virgin), sunflower seed oil, olives (green), peanut butter.
**Vegetables**	Onion (white), lettuce (green), arugula, asparagus, tomato (normal, cherry), spinach, globe artichokes, cucumber, zucchini, pumpkin, eggplant, Swiss chard leaves, endive, green beans, common cabbage green, sweet pepper (red, green), carrots, broccoli, cauliflower, celeriac, Brussel sprouts, capers, sweet potato (raw).
**Fruits and derivatives**	Apple, banana (raw), apricot (fruit, jam), pineapple, pear (peeled), orange, fig, peach, kiwi, lemon, mango, plum, tangerine, pomegranate, strawberry (fruit, jam), watermelon, honeydew melon, grapefruit, quince, guava, grape (green, raisin), bilberry, red raspberry (fruit, jam), avocado, custard apple.
**Processed**	Tomato sauce, ketchup, mayonnaise, cakes, cookies (normal, whole grain), potato chips snacks, vegetable mix (stew, cream).
**Condiments**	Cinnamon, pepper spice (black, white), vinegar, saffron, turmeric (dried), ginger (dried), cumin, nutmeg, cloves, anise herb (dried).

**Herbs**	Garlic (fresh), parsley (fresh), oregano (dried).
**Tubers**	Potato (boiled).
**Alcoholic drinks**	Beer, wine (red, white, rose), rum.
**Cocoa and derivatives**	Cocoa powder, chocolate (milk, dark).
**Infusions**	Tea (green), German camomile tea.
**Legumes**	Lentils, chickpeas, common bean (white).
**Nuts**	Almonds, walnuts, hazelnuts (raw), chestnuts, pistachio, peanut (roasted), sunflower seeds, cashew nut (raw), macadamia, brazil nuts.
**Juices**	Orange (pure juice), lemon juice from concentrate), pineapple (pure juice).
**Soy and derivatives**	Soybean sprout (raw), soy sauce, soy yogurt, soy milk.

Notes: Food items not found in the database and therefore excluded from the analysis: generic infusion, sesame seeds, flax seeds, mushrooms, honey, leeks, bay leaves, paprika, rice, almond and oat drinks, green peas, quinoa, beetroot, mustard, khaki, wakame seaweed, Christmas desserts (nougat, marzipan), palm oil, pickled cucumber, lamb's lettuce vegetables.

Table S2. Distribution of the daily dietary intake of (poly)phenols across the main food sources analysed (data obtained from 24h dietary recalls analysed by DIAL and Phenol Explorer database).

	N **(% of consumers)**	**Mean ± SD (mg/day)**	**Median (IQR) (mg/day)**	**[Min, Max] (mg/day)**	**CV(%)**
**Cereals and derivatives**	81 (100)	138.1 ± 67.6	136.4 (88.9)	[1.0, 330.5]	49.0
**Oils and olives**	80 (98.8)	15.6 ± 11.5	12.8 (8.6)	[1.0, 73.7]	73.3
**Vegetables**	80 (98.8)	119.4 ± 111.7	90.3 (99.9)	[5.8, 472,5]	93.6
**Fruits and derivatives**	74 (91.4)	293.2 ± 207.0	217.9 (311.8)	[1.3, 743.4]	70.6
**Processed**	71 (87.7)	48.9 ± 74.3	26.8 (39.2)	[2.2, 465.3]	>100
**Condiments**	64 (79.0)	21.6 ± 51.7	3.8 (8.2)	[0.2, 330.3]	>100
**Herbs**	62 (76.5)	2.8 ± 6.4	1.0 (1.6)	[0.2, 41.2]	>100
**Tubers**	61 (75.3)	49.0 ± 37.2	40.2 (48.0)	[4.5, 179.8]	75.9
**Alcoholic drinks**	52 (64.2)	131.3 ± 145.6	89.9 (166.9)	[0.4, 322.1]	>100
**Cocoa and derivatives**	46 (56.8)	371.0 ± 387.0	239.2 (312.5)	[14.5, 1645.6]	>100
**Infusions**	45 (55.6)	99.0 ± 63.7	82.3 (77.7)	[1.7, 247.4]	64.4

**Legumes**	31 (38.3)	504.5 ± 714.9	29.3 (1026.5)	[1.0, 3079.9]	>100
**Nuts**	28 (34.6)	216.6 ± 285.1	124.2 (259.5)	[2.6, 1398.0]	>100
**Juices**	24 (29.6)	42.0 ± 38.5	43.6 (50.9)	[2.7, 157.6]	91.5
**Soy and derivatives**	20 (24.7)	31.5 ± 36.4	18.9 (28.2)	[0.1, 118.2]	>100

N = Sample size of consumers of that food source. Percentage of participants that consume that food source in comparison with the total sample population included in the analysis (n=81).

Table S3. Component rotated array from PCA of the diet, eigenvalues, and percentage of total variance from each factor.

	PC1	PC2	Uniqueness
Energy Intake	**0.903**	0.349	0.062
Lipids	**0.896**	0.277	0.120
SFA [a]	**0.895**	0.139	0.180
Proteins [a]	**0.830**	0.155	0.287
MUFA [a]	**0.801**	0.282	0.279
Dietary cholesterol	**0.634**	-0.085	0.591
Carbohydrates	**0.623**	0.312	0.515
PUFA [a]	**0.615**	0.333	0.510
(Poly)phenols [a]	0.100	**0.881**	0.213
Dietary fibre [a]	0.252	**0.814**	0.274
Intrinsic sugars	0.161	**0.762**	0.393
Added sugars [a]	0.397	0.080	0.836
Eigenvalues	6.123	1.617	
Percentage of total variance	43.1	21.4	

Extraction method: principal component analysis using parallel analysis. Rotation method: Varimax. Superscript a indicated variables that were transformed using natural logarithms (Ln) to achieve a normal distribution before introducing them into the PCA.

Table S4. Component rotated array from PCA of anthropometric, body composition and biochemical measurements, eigenvalues, and percentage of total variance from each factor.

	PC1	PC2	PC3	Uniqueness
TG [a]	**0.860**	0.037	-0.014	0.259
VLDL [a]	**0.857**	0.037	-0.018	0.264
Waist/hip ratio	**0.764**	-0.182	0.065	0.379
HDL [a]	**-0.731**	0.072	**0.471**	0.238
HOMA-IR [a]	**0.684**	0.202	-0.184	0.457
SBP	**0.609**	-0.023	**0.427**	0.447
DBP	**0.560**	-0.028	**0.429**	0.502
BMI	**0.535**	**0.643**	-0.145	0.279
% Body fat	-0.069	**0.972**	0.087	0.042

	PC1	PC2	PC3	Uniqueness
% Muscle Mass	0.115	**-0.941**	-0.127	0.086
Visceral Fat Area	0.214	**0.932**	0.003	0.086
SUMM 6 skinfolds [a]	-0.137	**0.812**	-0.192	0.286
Total Cholesterol	-0.076	0.017	**0.914**	0.159
LDL	-0.028	-0.035	**0.853**	0.270
Eigenvalues	4.268	3.717	2.260	
Percentage of total variance (rotated solution)	29.4	27.5	16.2	

Extraction method: principal component analysis using parallel analysis. Rotation method: Varimax. Superscript a indicated variables that were transformed using natural logarithms (Ln) to achieve a normal distribution before introducing them into the PCA.

Table S5. Component rotated array from PCA of diet, anthropometric, body composition and biochemical measurements, eigenvalues, and percentage of total variance from each factor.

	PC1	PC2	PC3	PC4	Uniqueness
Energy Intake	**0.950**	0.021	-0.146	-0.051	0.072
Lipids	**0.921**	0.063	-0.043	-0.018	0.145
SFA [a]	**0.865**	0.041	0.011	-0.101	0.239
MUFA [a]	**0.839**	0.112	-0.016	0.089	0.275
Proteins [s]	**0.792**	0.187	-0.184	0.224	0.254
PUFA [a]	**0.702**	0.018	-0.066	0.104	0.492
Carbohydrates	**0.678**	-0.106	-0.166	-0.210	0.457
Dietary fibre	**0.570**	-0.094	-0.288	-0.198	0.545
Dietary cholesterol	**0.523**	0.294	-0.066	0.263	0.567
Intrinsic sugars	**0.510**	-0.094	-0.000	0.003	0.731
(Poly)phenols [a]	**0.487**	-0.191	-0.092	-0.039	0.716
Added sugars [a]	**0.411**	-0.232	0.113	-0.230	0.711
TG [a]	-0.148	**0.871**	0.006	0.006	0.220
VLDL [a]	-0.154	**0.868**	0.004	0.003	0.224
Waist/hip ratio	0.046	**0.764**	-0.164	0.033	0.386
HDL [a]	0.054	**-0.726**	0.077	**0.472**	0.241
HOMA-IR [a]	-0.031	**0.683**	0.176	-0.158	0.477
SBP	0.140	**0.589**	0.003	0.312	0.536
DBP [a]	0.182	**0.572**	-0.003	0.333	0.529
BMI	-0.089	**0.519**	**0.653**	-0.165	0.269
% Body Fat	-0.101	-0.086	**0.960**	0.114	0.047
% Muscle mass	0.107	0.126	**-0.926**	-0.159	0.090
Visceral Fat area	-0.155	0.194	**0.923**	0.016	0.087
SUMM 6 skinfolds	-0.111	-0.144	**0.790**	-0.150	0.321
Total cholesterol	-0.084	-0.035	0.042	**0.928**	0.129
LDL	-0.058	0.013	0.007	**0.868**	0.243
Eigenvalues	6.734	4.394	3.431	2.440	
Percentage of total variance (rotated solution)	24.0	16.8	15.2	9.4	

Extraction method: principal component analysis using parallel analysis. Rotation method: Varimax. Superscript a indicated variables that were transformed using natural logarithms (Ln) to achieve a normal distribution before introducing them into the PCA.
